# A study skills course for First-Year Medical Students: Experience of a Private Medical School in Pakistan

**DOI:** 10.12669/pjms.37.1.2772

**Published:** 2021

**Authors:** Sobia Ali, Afifa Tabassum, Muhammad Suleman Sadiq Hashmi, Nighat Huda

**Affiliations:** 1 Dr. Sobia Ali, MHPE. Department of Health Professions Education, Liaquat National Hospital and Medical College, Karachi, Pakistan; 2 Dr. Afifa Tabassum, MHPE. Department of Health Professions Education, Liaquat National Hospital and Medical College, Karachi, Pakistan; 3 Dr. Muhammad Suleman Sadiq Hashmi, MHPE. Department of Health Professions Education, Liaquat National Hospital and Medical College, Karachi, Pakistan; 4 Prof. Nighat Huda, MSED. Department of Health Professions Education, Liaquat National Hospital and Medical College, Karachi, Pakistan

**Keywords:** Study Skills, Coping Strategies, Academic Stress, Undergraduate Medical Students, Postgraduate Medical Students

## Abstract

**Objectives:**

To share the experience of study skill module development and implementation for first year MBBS students at Liaquat National Hospital and Medical College (LNH&MC). To compare the change in students’ self- assessment of their knowledge of study skills before and after the course.

**Methods:**

This quasi-experimental study was conducted from November 2019 to April 2020. A structured study skills course was offered to 100 first year MBBS students of Liaquat National Hospital & Medical College, Karachi. Steps involved in the development included identification of outcomes, instructional objectives, content and instructional strategies. Students were given two questionnaires. In the first questionnaire, students rated their interest in implementing the learning techniques learnt. In the second questionnaire, students rated their level of knowledge of effective study skills before and after the course. Analysis included computing percentages for students’ preferred study skill technique. Change in knowledge was assessed by comparing retrospective pre-post self-rating using Wilcoxon Signed Ranks Test (two-tailed).

**Results:**

Analysis of survey forms showed that more than 50% of the students were willing to implement active listening techniques, metacognitive note taking and writing reflections in their future study practice.There was also a statistically significant change in students’ self-rating of their knowledge about study skills (pre-test median 3, post-test median 4, p0.00).

**Conclusion:**

This study provides an insight of structured study skills course development and implementation in early medical college studies that could help them in combating academic stress. In addition, students’ response about their preferred technique and their feedback comparison concluded their positive attitude towards the course.

## INTRODUCTION

The transition from relatively stable and didactic teaching at high school to the strikingly different reality of medical school is challenging for medical students worldwide. The significant amount of educational content and duration to understand it is inversely proportional, leaving students frustrated and calling for study skills support.^1^ It is often stated that study skills that made students successful in high school turn out quite insufficient and inefficient for success in medical school.^2^ Students realize that the overwhelming burden of information needs to be addressed and requires certain coping strategies. It is also evident that those whoface difficulty in coping, come under huge academic pressure, resulting in their poor academic performance.^3^

Academic pressures are the top most stress inducers among medical students with resultant academic failure.^4^ To address this stress, medical schools offer informal support by more approachable faculty and structured mentoring programs. Beside these supports, students need help in improving study habits, managing time effectively and efficient techniques to improve their study skills.^5^,^6^ Structured study skills courses are thus required to improve academic performance and to alleviate academic stresses.^7^ Though some medical schools have study skills programs for undergraduate as well as postgraduate students, there is insufficiency of evidence regarding structured study skills programs in our context.^7^,^8^ Huda and Burla in 1999, shared their experience of study skill module development with respect to the background of implementing problem-based learning (PBL) to acquaint students with small group dynamics.^9^ Although studies from non-med specialties are available, there is a dearth of literature on study skills courses for medical students in Pakistan.^10^,^11^ Competencies required for non-medical graduates vary from medical graduates and therefore a structured study skills course for medical students is recomended to deal wih academic stress and performance anxiety.

Liaquat National Hospital & Medical College (LNH&MC) caters admissions of applicants from a diverse socio-economic and educational background of Pakistan. This diversity adds to the educational stress on first year medical students.^7^ Department of Health Professions Education (DHPE) at LNH&MC decided to help newly inducted medical students to prepare them better for the upcoming academic stress. For this, we developed a structured study skill course to help medical students build effective study skills to cope better with academic stress. We were also of a view that carefully planned and implemented study skills courses at the commencement of undergraduate medical training can go a long way in helping to prepare the medical students, not only for the rigors of undergraduate study but for a lifetime of continuing education and practice. With this newly developed course,the aim of this study is to share the experience of developing study skill module for first year MBBS students including the content, instructional strategies, and students’ preferences for specific skills. We also wanted to compare the change in students’ self- assessment of theirknowledge about study skills before and after the course.

## METHODS

This quasi-experimental study was conducted from November 2019 to April 2020. The study was approved by Ethical Review Committee (Ref: APP # 0515-2020-LNH-ERC, Dated April 23, 2020) of LNH&MC, Karachi.

### Course development

DHPE formulated a planning committee comprising four members to develop the course. Once the outcomes were finalized, the committee further elaborated it by listing instructional objectives and contents. Instructional strategies were then decided using guidelines from large body of literature on active learning techniques (Table-I). An 8-hour-course was then developed and offered to 100 students of first-year MBBS during their foundation module in February 2020.

**Table-I:** Study skills course sessions’ title and instructional strategies

S.No.	Title	Duration	Instructional strategy	Proposed timein foundation module
1.	Know your Learning Style	1 hour	Large group exercise	Within First 2 days of foundation Module
2.	Learning experiences and test-taking skills	1 hour	Senior students led seminar	1^st^week
3.	Effective Study Skills of Successful Students-1	2-hour	workshop	1^st^ week in small groups
4.	Effective Study Skills of Successful Students-2	2-hour	workshop	2^nd^ week in small groups
5.	Understanding the science of lifestyle to improve your study-1	1-hour	Interactive lecture	3^rd^ week
6.	Understanding the science of lifestyle to improve your study-2	1-hour	Interactive lecture	4^th^ week

This course was divided into four components that were taught in six sessions during the 1^st^ month of study at LNH&MC. Out of these six sessions, two sessions were conducted in a workshop format, one was student led seminar, one was a large group exercise, and two were interactive lectures (Table-I).

### Instructional strategies:

### Session 1: Know your learning styles:

During this one-hour session, the concept of learning styles and its effect on learning was introduced to the students. Students were given the Approaches and Study Skills Inventory for Students (ASSIST)^12^ to identify their predominant learning approach. Importance of being aware of one’s learning style and its utilization in adopting different learning techniques to be taught later on was then discussed. This was purely a self-evaluative exercise for students and no analysis of the student scores of the inventory was done to this point.

### Session 2: Learning experience and test taking skills

This one-hour seminar was led by senior students who have passed their Year-1 MBBS and achieved high grades in their university exams. These students first shared their learning experience and strategies that helped them achieve high grades. After that, main focus was on an informal discussion on study hours, study style, test-taking skill, study organization, preference for group study or not, resources to be used(reference books, guide books, internet).

### Sesssions 3 & 4: Effective study skills of successful students–(2 workshops)

The 2-hour workshop format was used for these sessions (25 students per session). Two workshops were developed. These workshops were an adaptation from MedEd portal resources for study skill with modifications to make it adaptable in our context. This resource consisted of instructional tools for faculty to support the remediation of learners with suboptimal study habits, based on adult learning theory.

Course director (SA), took permission from Rodney L Nyland (corresponding author for this open access resource) on email to use this resource for our students. Facilitated by DHPE faculty, these workshops were designed to make students identify the weaknesses in their learning approaches and strategies and to equip them with more effective study skills like chunking, spaced practice, metacognitive note taking and reflective writing. To reinforce the concepts given during the workshops, a handbook was also given to students with focus on strategies that can be used before, during and after the lectures to improve their learning.

### Sessions 5 & 6: Understanding the science of lifestyle to improve your study

Two interactive lectures in large class format were conducted by an expert Psychiatrist who guided students on copingwith pressures of medical school by adopting a healthy lifestyle. The content of the sessions also included stress management and time management strategies in relation to the lifestyle modification.

### Data Collection

Students were given two questionnaires. The first questionnaire was given immediately after the workshops in which all the learning techniques discussed during the workshops were listed and students were asked to rate their interest in implementing each technique. The level of interest ranged from ‘I am not interested in this technique’ to ‘I will implement this technique’. Students’ interest was measured in terms of percentages for each technique taught in workshops. This questionnaire was also adapted from MedEd portal resources for study skill.^13^ with slight modificationsto fit better with the content of our workshop. Statement about *writing reflections* was added in the survey because we made our students to practice reflective writing during the session. Similarly statements about hand *wrting notes* and and *rewordin/rephrasing the lecture content* were ommitted because of not being dealt and emphasized during our workshop

**Fig.1:**
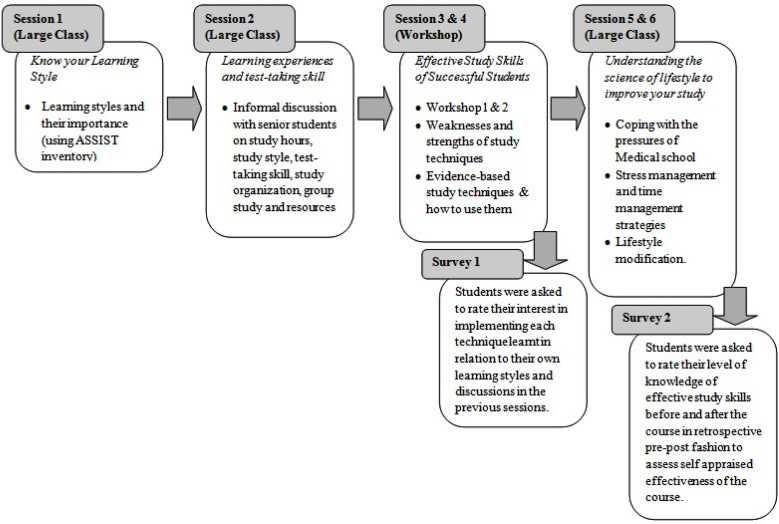
Sessions and sequence of data collection during study skills course

Second questionnaire (developed by Course Director and reviewed by three medical educationists) was distributed after the last session to get the feedback of students using surveyplanet. It comprised of statements regarding the knowledge about the content of the course. Students were asked to rate their level of knowledge of effective study skills on the scale 1-5 (where one being very low and five being very high), before and after the course in a retrospective pre-post fashion. We use this approach because it prevents the response shift bias presented in traditional pre-post design.^14^ Sessions and sequence of data collection is represented in Fig.1.

### Statistical Analysis

Data was analyzed by SPSS version 20. Analysis methods included computing percentages for students’ preferred study skill technique. Change in knowledge was assessed by comparing the responses in retrospective pre-post self-rating using Wilcoxon Signed Ranks Test (two-tailed). This non-parametric test was used because of skewed data.

## RESULTS

After development and implementation of the study skills module as described above, the students’ preferences for specific skills was measured through the first questionnaire. With 80% response rate, (n= 80 out of 100 First year students) our first survey showed students’ level of interest in implementing each technique discussed in the workshop (Table-II). 45% students stated “test myself” as a strategy that they previously used and will continue to use.More than 50% of the students were willing to implement active listening techniques, metacognitive note taking and writing reflections in their future study practice.

**Table-II:** Students’ level of interest about study techniques (n= 80)

S.No.	Study Skills Technique	*I am not interested in this technique*	I previously used it and it didn’t work for me	I previously used it and will continue to use it	I am interested in this technique but need more information	I will implement this technique
	Before the class					
1	Active reading	13%	4%	31%	21%	35%
2	Preview session objectives	13%	9%	18%	15%	46%
	*During the class*					
3	Active listening	3%	9%	31%	15%	51%
4	Metacognitive note taking	11%	3%	11%	23%	55%
	*After the class*					
5	Create own study materials	14%	10%	29%	18%	31%
6	Making connections	6%	3%	41%	16%	35%
7	Reviewing with objectives	5%	6%	39%	15%	35%
8	Spaced retrieval	23%	8%	34%	15%	21%
9	Test myself	4%	4%	45%	13%	38%
10	Writing questions	10%	6%	30%	11%	43%
11	Writing reflections	4%	2%	23%	34%	58%

The change in students’ self- assessment of their knowledge about study skills before and after the course was evaluated through the second questionnaire. Response rate was 81% (n=81out of 100 First year students). Using Wilcoxon Signed Ranks Test (two-tailed), we found a statistically significant change in students’ self-rating of their knowledge about study skills (pre-test *median* 3, post-test *median* 4, p-0.00). In addition, the trend of higher ratings by students is prominent on all items after the skills course (Table-III).

**Table-III:** Comparison of retrospective pre-post self-ratings of students by Wilcoxon Signed Ranks Test

*S.No.*	*Statements*	*Negative Ranks*		*Positive Ranks*		*p*-value

		Mean	Sum of Rank	Mean Rank	Sum of Rank	Rank
1	I am able to identify my predominant learning approach	26.4	132	32.48	1884	0.000*
2	I am able to differentiate among low and high utility learning strategies	29.21	204.5	35.11	2141.5	0.000*
3	I am aware of the strategies that I can use before the lectures for improved learning	21.83	65.5	27.83	1419.5	0.000*
4	I am aware of the strategies that I can use during the lectures for improved learning	21.25	85	25.33	1140	0.000*
5	I am aware of the strategies that I can use after the lectures for improved learning and retention	23.39	210.5	31.75	1619.5	0.000*
6	I know how to take metacognitive notes during the lectures	29.25	58.5	30.03	1711.5	0.000*
7	I know how to write reflection after the lectures	12.5	37.5	29.41	1558.5	0.000*
8	I am aware of effective test taking skills	16	16	25.69	1259.0	0.000*
9	I am knowledgeable about healthy study habits	14	28	24.44	1100	0.000*

## DISCUSSION

With the continuous explosion of new knowledge, medical education strives to focus on learners and facilitating learning.^15^ Unfortunately, most medical schools are lacking in providing structured support to enhance study skills.^16^ This course in some schools is limited to the identification of students’ individual learning styles, study skills, and approaches to learning by using inventories.^17^-^19^ In this study, we share our newly developed structured course to enhance study skills for first year medical students. Taking guidance from literature, we tried multiple strategies that have shown tooffer the greatest impact. While covering multiple aspects of skill required for performing well in medical school, this course can help students to combat academic stress.

Results of students’ preference of specific style showed that students are willing to apply those techniques more that were practiced well during the session. There was a designated time and structured activities for metacognitive note taking, active listening and reflective writing practice. The rest of the techniques that can be used to enhance study skills were delivered in the form of power-point presentations and handouts. This seconded the literature claims that strategies in which the students are actively engaged result in better learning and enhances interest in the subject.^20^ This also verifies the findings by Bajwa et al. that students with formal training are significantly better on note-taking.^10^

Evaluators of short courses usually include pre and post self-rating to determine course effectiveness. In this study, we used retrospective pre-post assessment to avoid response shift bias and to provide more accurate measurement of their baseline knowledge.^21^ With this baseline assessment, the retrospective pre-post survey findings showed that students thought that their knowledge about various aspects of study skills was highly increased (p=0.000*). They agreed that their knowledge of effective test taking skills was improved and that they are able to differentiate among low and high utility learning strategies better. This is in accordance with the findings of previous study of Cynthia J Miller that claimed that structured study skill course help improve test-taking skills.^8^ Similarly, students clearly stated that this course helped them know the strategies to be used before, during and after the lecture better. This supports the positive feedback given by the students in a study by Siddiqui and colleagues.^7^ Knowledge about healthy lifestyle also increases after attending the course supports the previous evidence.^22^-^24^

### Limitations of the study

Our feedback data comprised of self-rating of students and may not reflect the true approach of students’ learning. Other limitations include single cohort study and comparing the students’ self- rating on a small range of course content. However, given these limitations, we felt that there is a need to publish this study because it will provide initial direction and approach to develop such courses, especially in Pakistani context. Ongoing research by the authors on the assessment of study techniques and its impact on students’ learning will further elaborate the content.

## CONCLUSION

The present study primarily focused on the development of study skill course for first-year medical students. After going through the process of developing and later conducting this course, and the analysis of students’ feedback, the high level of students’ satisfaction can be easily concluded. Further, with this level of satisfaction, this course is likely to assist 1^st^ year MBBS students in improving their learning strategies and skills. This study can be considered as a baseline study that could give future direction for the development of such courses for undergraduate as well as postgraduate medical education.

### Authors’ Contribution:

**SA:** Conceived the idea, designed research, analyzed data, drafted manuscript and responsible and accountable for the accuracy and integrity of the work. **AT, SSH:** Collected the data, contributed in research design and manuscript revision. **NH:** Contributed in research design, edited and revised manuscript.
